# Doctor of Science (D.Sc.): Time to move towards Higher Doctorate Degrees

**DOI:** 10.12669/pjms.37.7.5119

**Published:** 2021

**Authors:** Sultan Ayoub Meo, Shaukat Ali Jawaid, Nadia Naseem

**Affiliations:** 1 Sultan Ayoub Meo, MBBS, Ph.D. (Pak), M Med Ed (Dundee), FRCP (London), FRCP (Dublin), FRCP (Glasgow) FRCP (Edinburgh), Professor and Consultant, College of Medicine, King Khalid University Hospital, King Saud University, Riyadh, Saudi Arabia. Email: smeo@ksu.edu.sa sultanmeo@hotmail.com; 2 Shaukat Ali Jawaid, Chief Editor, Pakistan Journal of Medical Sciences, Secretary, Eastern Mediterranean Association of Medical Editors (EMAME). Karachi, Pakistan; 3 Nadia Naseem, MBBS, M.Phil, Ph.D. Professor, Department of Morbid Anatomy and Histopathology, University of Health Sciences, Khayaban-e-Jamia, Lahore, Pakistan. Email: headpathology@uhs.edu.pk

**Keywords:** Higher Doctorate, Doctor of Science, D.Sc, Academic title

## Abstract

The present most modern and highly advanced 21’st century is the era of science and technology. In human history, universities are the basic birthplace of higher education, research, and innovation and play a significant role in the countries’ performance, prosperity, and economic progress. Worldwide, there is a swift shift in the pattern of biological, environmental, economic, and educational systems. This broader change is rotating around the higher academia and its allied innovative research impact. The leading universities develop a culture and curricula as per need and demand and produce knowledge and skills-based professional graduates. The universities prepare graduates to keep in view their country’s requirements and compete with their peers at international levels.

Moreover, worldwide, universities are transforming towards higher doctorate degrees (D.Sc / S.Dc) to provide an elevated helipad to the applicant to compete in this modern and highly advanced era. The higher doctoral degree, D.Sc, is earned 6-8 years after the post Ph.D. The candidates with higher academic titles, professional skills, and innovative research could compete and achieve top-ranked positions worldwide. Many universities worldwide, including the United States of America, the United Kingdom, Australia, and New Zealand, promote D.Sc degree programs in various science disciplines, including medical sciences. This manuscript explores the dynamics of a higher doctorate and its significance, need, and demand in academia to compete globally.

Worldwide in higher education, competition is a comparatively old phenomenon but has new diverse dynamics that greatly influence the nations’ health, public prosperity, economies, and sustainable development. Higher Education has a strong association with the global knowledge economy and its influence on globalization.^1^ The international competition in academia is fueled by the idea of the “world-class university”.^2^,^3^ where renowned faculty and scientists are engaged in producing innovative research, thus raising the global rankings of the universities.^4^ The authors of this manuscript further coin this concept that “best universities are those which produce evidence-based, research-oriented five-star physicians, scholars, and scientists who are practicing high levels of research, ethics and professionalism.”

In recent decades worldwide, there has been an accelerating pace in developing and adopting novel technologies, although gaps persist in various corners of the globe, particularly in developing countries.^5^ This swift technological change affects every segment of life, including the economy, society, and culture.^5^ The academic and technology-based change has also moved around the higher academia and innovative research.^6^ The world today is prepared for another significant shift in the understanding of the university as an institution. The nations with high literacy rates, mainly higher education, strive towards academic excellence, scholarship and continuous progress, and development to compete globally.

In the global competition, the role of universities is enhanced to provide sub-specialty-based academia and research opportunities for their graduates to compete and achieve leading positions with sustainable development. In the 1960s, simple university graduates could get a good place in any national and multinational organization. In the 1970s, the graduates were gradually replaced by postgraduates. However, in the early 1980s, the doctorate Doctor of Philosophy (Ph.D.) became a more popular and terminal academic title. The Ph.D. scholars were moved towards a postdoctoral experience known as “post-doctorate or postdoc.” A postdoc is a 6-12 months research position that allows Ph.D. scholars to enhance their research skills and knowledge and prepare them for their progressive academic careers. Although, there is no official degree awarded for postdoc training.

In this science and technology-based modern era, there is a significant change in every segment of life, including academic titles in various science disciplines. The academicians and researchers are moving fast towards higher doctorate educational titles, Doctor of Science. The higher doctorate, “Doctor of Science,” also known as “D.Sc or Sc.D,” is a postdoctoral degree program that is awarded to an individual who has significantly contributed to the field of research and science. (Table-I) Many universities award this degree based on the extensive research work published in reputable science journals. (Table-II-III) After Ph.D., the D.Sc could be awarded to a candidate who has vast experience in higher academia, research and earns excellence as an academician and a researcher in a specific field. The main advantage of acquiring a D.Sc degree is that this title could provide a high helipad to the applicant to compete in this modern and highly advanced era and get a leading position globally. It also provides a chance to excel at broader levels in a particular specialty. Moreover, the benefits are limited not only at personal levels but also at institutional and state levels. The science community believes that this title can better contribute to knowledge-based economies.

**Table I:** Comparison between Doctor of Philosophy (Ph.D.) and Doctor of Science (D.Sc / S.Dc) Degrees.

*Characteristics*	*Doctor of Philosophy (Ph.D.)*	*Doctor of Science (D.Sc / S.Dc)*
Abbreviation	Ph.D. Doctor of Philosophy	DSc. Doctor f Science
Awarding body	University	University
Qualification Required	Postgraduate degree with course work, research, and thesis.	Post Ph.D. degree. Excellence in research, outstanding portfolio with academia, and published research
Duration required	Approximate 4-5 years after master degree	Approximately 6-8 years after post Ph.D.
Examination-based OR Honorary	Research-based The award may also be honorary	Excellence in academia and research The award may also be honorary
Field specified	Any field including, science, social sciences, medical sciences	Specified in Science subjects
Examination pattern	Evaluation of coursework, research, and thesis	Evaluation of academic and research credentials through external evaluators
Ranking status	Ph.D. is less prominent than D.Sc	DSc is a higher doctorate superior to PhD
Outstanding status	A routine academic degree	Elite qualification, academic title
Highest award conferred	To some extent highest recognition by the university	Highest distinguished award by the university
Job applications	Academia, research, corporate, organizations, etc.	Leading position in academia, research, corporate, organizations, etc.
Carrere scope	Carrere starts with the junior position, including lecturer / Assistant Professor, etc.	Carrere starts with senior positions, mainly Professor, Director, Head of the Institute, Dean, Rector, etc.
Perks, prestige, and privileges	Suitable	Supreme

**Table II:** Higher doctorate (DSc / S.Dc) degrees awarded in medical science by various universities in the United Kingdom.

*University/ Institute*	*Rank*	*Eligibility*	*Exam Format*	*Basic Sciences / Clinical*	*Scrutiny levels*	*No. of Examiners*
Imperial College London	8	Post Ph.D. 5 years	Published research work	Both	Special Committee	3 External
University of Birmingham	92	6-7 years Post Ph.D./ fellowship	Published research work	Both	Special Committee	3 External
Ulster University	601	Ph.D. 6 years	Honorary Published work	Clinical / Basic sciences	Research Board / Dean	2 External One internal
University of Bristol	86	Post Ph.D. 5 years Six years post-fellowship	Honorary / Published research work	Both	Research /Director	2 External One internal
University of Leeds	160	Post Ph.D./ Fellowship 6 years	Published research work	Both	Research division	Examining panel
University of Oxford DSc / DSc	5	Ten years post qualification	Published research work	Med Sci	Research panel	2 External
University of Southampton	90	Six years post Ph.D. Fellowship	Published research work with /VIVA	Both	Research panel	Two external
University of Warwick	66	Post PhD/M.S/ 10 years	Published research work	Clinical/ Basic sciences	Research panel	One Internal One external
University of Manchester	51	Ph.D-07 years Ten years after Ten years after MS/FellowshipMS/Fellowship	Published research work / VIVA	Both	Research panel	3 External
University Sheffield	93	Ph.D. 6 years	Published research work	Both	Research panel	2-3 All external
Queens University Belfast	21	Ph.D. 10 years	Published research work	Sciences	Research panel	Two external One internal
Durham University	87	Post Ph.D. 4 ys, MS/Fellows7 ys	Published research work	Both	Research panel	Two external One Internal
University of Exeter	149	Post Ph.D. 07 years	Published research work with VIVA	Sciences	Research panel	Examination Committee
University of Liverpool	122	Post Ph.D./MSc with 07 years	Published research work	Sciences / Medical	Research panel	Two external evaluators
Monash University	55	Post Ph.D. 7 years	Published research work	Both	Research panel	3 External
Nottingham University	103	MS/MSc/Ph.D 7-8 years	Published research work	Both	Research panel	Total 2-3 ll external
National academy of sciences	----	Post Ph.D. 10 Fellows of academy ten years	Published research work	Basic / Biology Sci	Research panel	Same board
Harvard School of Public Health	3	Ph.D. 10 years Fellows of academy ten years	By thesis	Both	Dean only	Two external One Internal

**Table III:** Higher doctorate (DSc / S.Dc) degrees awarded in medical science by various universities in Asian Universities.

*University/ Institute*	*Rank*	*Eligibility*	*Exam Format*	*Basic Sciences / Clinical*	*Scrutiny levels*	*No. of Examiners*
University of Karachi		15 years post-PhD MD/MS	Published research work / Honorary	Science	Advanced Studies and Research Board (ASRB)	Three external Evaluators
COMSAT University Islamabad		15 years post-PhD MD/MS	Published research work / Honorary	Sciences	Advanced Studies and Research Board (ASRB)	Three External Evaluators
Sri Ramachandra Institute of Higher Education and Research, Chennai India	----	15 years post-PhD MD/MS	Thesis	Basic and Clinical Medical Sciences	Higher Doctoral Committee	Two external One internal
University of Madras, India	----	Post Ph.D. post years MS/MSc 15 years	Thesis	Sciences	Special Committee	3external Evaluators
Aliah University, India	-----	Post Ph.D./MSc with 05 years	Thesis	Sciences	Research Board	5-6 external evaluators
University of Nairobi, Kenya	650-750	Post Ph.D. with 05 years	Published research work	Medical Sciences	Special Committee	Examination Committee
University of Peradeniya	402	Post Ph.D. with a substantial contribution	Published research work	Medical Sciences	Director Research	Examination committee
National academy of sciences	----	Post Ph.D. 10 years, Fellows of academy 10 years	Published research work	Basic / Biology Sci	Board of Research	Same board

Worldwide, like other higher academic titles, universities are awarding this title through two routes, research work and honorary. Table-II-III. The Doctor of Science degree offers various opportunities in terms of professional career. This title is pursued to recognize an applicant’s work and advanced research skills and provides opportunities for candidates to work with global universities, research centers, and organizations. The higher doctorate academic titles facilitate the topmost positions in the leading national and international institutes and organizations. The career opportunities, perks, prestige, and privileges of D.Sc degree holders are significantly better than Ph.Ds (Table-I).

In the new millennium worldwide, there is a significant shift in the pattern of biological, environmental, economic, and educational systems. This broader change and its impact is rotating around the higher academic titles and innovative research. The universities are developing the curricula and producing graduates according to the community’s needs and international demand. Moreover, the universities prepare their graduates not only to compete with their peers at national levels but also at international levels. The universities are transforming their academic framework towards higher doctorate degrees at a fast pace (DSc/S.Dc).

In many countries, the D.Sc, a higher doctoral degree, is earned a few years (about 6-8 years) after the Ph.D. Once their graduate has higher academic titles with published innovative research, they can easily compete for top-ranked positions at global levels. The United States of America, the United Kingdom, Europe, Australia, New Zealand, Russia, India, and few universities in Pakistan promote higher doctorate in various science disciplines. Table-II-III.

In human history, the universities, academic and research institutes have produced scholars who compete at international levels and deliver beneficial impacts to societies. Higher Education is the right path to the empowerment of people and the sustainable development of nations. It is a fact that advanced countries have focused primarily on the progressive academic institutions that would facilitate knowledge creation and dissemination. The advanced universities encouraged sub-specializations structured in the various disciplines. However, a growing awareness has appeared that the difficulties of the 21st century require a holistic experience of knowledge in its various aspects. Presently, the gauges of strong and sustainable economies depend on advanced academia and innovative research. Research-based higher academia plays a vital role in the nations’ excellence of life, economies, and sustainable development.^7^

Pakistan is home to “224 million people^8^ and 229 universities,^9^ including 30 medical universities, 176 public and private sector medical and dental schools’^10^ 125 engineering, 98 management sciences, and 30 agricultural institutes”. It is high time that Pakistan must understand that the survival and ranking of its research universities in the coming decades depend upon the provision of advanced education and research qualification with a significant contribution towards the complex, globalized economies of the 21st century.

National competition and global competition are two distinct domains; higher education provides social prestige and income-earning access. The global competition in higher education is an emergent property of competitive relations among nation-states. The new institutionalist approach claims that the international competition results from the universities turning into organizational performers^1^. The universities compete in the global higher education market. To achieve a higher competitive position at international levels, the candidate must attain the highest academic titles. Similarly, the students and faculty must achieve unique academic titles to compete at international levels.

As academic commercialization progresses, universities must operate similarly to multinational corporations. Indeed, not all universities participate in the global competition, but only those with a worldwide capacity “world-class university”.^11^ Similarly, not all students, researchers, and faculty members participate in the global competition, but only those who have the potential to earn the highest possible academic titles and research to become a “world-class researcher or faculty.” The best universities produce well-qualified, evidence-based, research-oriented graduates who are in high demand in the intellectual and skill-based market; they conduct leading-edge research published in the top scientific journals, and contribute to innovations through patents and licenses.”^2^,^3^,^11^

Narrating about Pakistan, we have the shining example of Aga Khan University, which attracts numerous research grants worldwide, has earned a name, and established its credibility as a leading university. Though they enjoy numerous advantages, this does not mean that public sector medical universities may not strive for excellence. In fact, the problem with us in the field of higher education and higher medical education, in particular, is that Higher Education Commission which looks after higher postgraduate education, neither has a separate section or division of medical education nor anyone with the medical background; hence it is futile to expect any revolutionary steps from it in this field. We had pointed out even earlier^6^ that we need to establish a separate division of higher medical education within HEC headed by a qualified medical educationist with proven academic accomplishments for better understanding and resolution of these issues with a vision to plan and implement policies aimed at facilitating reforms at our public sector medical universities. Ideally, it would be much better if the higher medical education is shifted from HEC to the Ministry of Health and Medical Education by changing its name as was done by one of our neighboring countries in 1985, and it paid rich dividends.

Yet another problem that we face in Pakistan is the selection of Vice-Chancellors to head these universities, which is most often based not on merit but some other considerations. Of course, there are exceptions when people have been selected on merit. The rot starts here. Those who feel their rights have been ignored then go to the courts to seek justice and suspend such orders.^12^ Incompetent, inefficient, ill-trained, those who lack foresight and vision when appointed to these coveted posts themselves feel threatened. They do not encourage competent and highly qualified people among the faculty but feel secure while surrounded by “Dead Wood,” who will sing their praises all the time. Again, we have not yet understood the difference between the post of the Dean, Principal, and the Vice Chancellor. These posts have different rights and responsibilities. However, in Pakistan, many medical universities do not have the post of the Dean and or Principal. Those who inserted these clauses in the Act did not want to share the powers and wanted themselves to be fully powerful; little do they realize that they are not going to be in those posts forever, and graveyards are full of people who once thought themselves indispensable. The first and foremost important thing is to make necessary changes in the Act that help establish these universities, having Principal and clearly laying down the rights and responsibilities of each one.

The Principal, the Dean, looks after the medical student’s affairs and issues related to undergraduate medical education, whereas the Vice Chancellor plans to start a new postgraduate academic programme, research projects, arrange funding through different sources, and establish international linkages with institutions of excellence in the respective fields. With the team’s help, schemes to generate funding enables the university to reduce its dependence on government funding. If there is a will, there is always a way. Just look at Dow University of Health Sciences and Jinnah Sindh Medical University in Karachi. Both have established a state-of-the-art Research and Reference Laboratory. The DUHS, in particular, established a network of collection units all over the city and earns a lot of money which is an additional useful resource for these universities. Why cannot this be replicated and what forbids the Vice Chancellors of other public sector medical universities to opt for this model? Faculty infected with politics effectively retards the growth and development of our medical universities. The working environment is such that the public sector medical universities also fail to attract and retain talented faculty for reasons that are not difficult to understand.

It is extremely important that to gain a competitive advantage, universities must launch novel, specialized degree-awarding programs so that their graduate could find a place in a global market. They have to engage their graduates and faculty in innovative research, patents, profit-oriented academic activities. It is high time to appreciate the worth of higher education, innovative research, and its impact on socio-economic development and political stability. Pakistan should implement strict policies to establish and promote higher postgraduate degree programs, Ph.D., and DSc. The advanced higher academic titles facilitate the graduates to compete with the world and lead the state towards a knowledge-based economy.^6^ Higher education is the only weapon of any nation to fight against poverty, terrorism and bring peace, prosperity, socio-economic development, and political stability in the country and the region.

The University of Health Sciences (UHS), Lahore, has started multiple novel postgraduate programs in basic and clinical medical sciences, medical education. More recently, it has started a Certificate Course in Medical Editing, which will eventually lead to Masters in Health Journalism. It will comprise four Modules of six months each. 13 Pakistan happens to be the second country in the EMRO Region to have started this innovative program to train the medical editors, which will help improve the standard of medical journals published from Pakistan.

The UHS, in its adolescent age, has produced many Ph.Ds in various disciplines of medical sciences. Furthermore, UHS leads in launching a higher doctorate degree program, D.Sc in medical sciences. These graduates will play a significant positive role in academics, research, innovation and benefit humanity and the country. Therefore, Pakistan must set the higher education reforms and research-oriented architecture that can emerge as a knowledge power.^6^
